# Dissolved Organic Carbon Mobilisation in a Groundwater System Stressed by Pumping

**DOI:** 10.1038/srep18487

**Published:** 2015-12-22

**Authors:** P. W. Graham, A. Baker, M. S. Andersen

**Affiliations:** 1 Connected Waters Initiative Research Centre, UNSW Australia, Sydney, NSW 2052, Australia

## Abstract

The concentration and flux of organic carbon in aquifers is influenced by recharge and abstraction, and surface and subsurface processing. In this study groundwater was abstracted from a shallow fractured rock aquifer and dissolved organic carbon (DOC) was measured in observation bores at different distances from the abstraction bore. Groundwater abstraction at rates exceeding the aquifers yield resulted in increased DOC concentration up to 3,500 percent of initial concentrations. Potential sources of this increased DOC were determined using optical fluorescence and absorbance analysis. Groundwater fluorescent dissolved organic material (FDOM) were found to be a combination of terrestrial-derived humic material and microbial or protein sourced material. Relative molecular weight of FDOM within four metres of the abstraction well increased during the experiment, while the relative molecular weight of FDOM between four and ten metres from the abstraction well decreased. When the aquifer is not being pumped, DOC mobilisation in the aquifer is low. We hypothesise that the physical shear stress on aquifer materials caused by intense abstraction significantly increases the temporary release of DOC from sloughing of biofilms and release of otherwise bound colloidal and sedimentary organic carbon (SOC).

The concentration of DOC in groundwater is highly variable and sources and migration mechanisms are poorly understood. In soils and riverine systems, sorption and desorption of SOC from sediment and colloids, photo-degradation in the aquatic photic zone, and biodegradation are established factors that control OC biological and physiochemical processing^1^,^2^,^3^,^4^. Studies of riparian groundwater have described rivers as a major source of autochthonous and allochthonous DOC during periods of infiltration^5^,^6^,^7^. In addition subsurface flows that include groundwater interactions with the soil horizon, vegetation, or buried carbon lenses can cause significant variations in DOC distribution and concentrations in terrestrial waters^8^,^9^. Attenuation of DOC in soils is understood to be directly controlled by adsorption, co-precipitation and biodegradation. Groundwater redox state, pH, nutrient availability, and water movement through the soil profile are thought to indirectly influence DOC attenuation^10^.

Research into the stabilisation and destabilisation of groundwater OM has focussed on the effects of: biofouling due to injection of nutrient rich water during bioremediation of contaminated soils or groundwater^11^,^12^,^13^,^14^,^15^,^16^,^17^; the creation of impermeable barriers around contaminated sites using enhanced biofilm growth^18^,^19^,^20^; the injection of water into oil reservoirs^21^; injection of CO_2_ rich brine on biofilm growth^22^. Typically these studies identify a significant accumulation of aerobic microbial populations in the immediate vicinity of the injection well, with increased concentrations of anaerobic microbial populations in areas further from the well screen^17^,^21^. Biofouling reduces the aquifer hydraulic conductivity, and therefore the majority of work undertaken focusses either on modelling the biofilm accumulation^22^,^23^,^24^, or replicating the systems in laboratory experiments to determine the most effective method for removal or reduction of the biofilm^14^,^15^. This existing literature is focussed on introduction of fluid to a porous medium (generally unconsolidated material or sandstone), which results in increased microbial growth either as an accidental or a planned occurrence. We are not aware of any research on OM accumulation and removal in fractured rock aquifers and none that considers the effects of intense groundwater extraction on native DOM.

Research into potential variations in DOC mobilisation during abstraction are limited to studies of the role of terrestrial organic carbon in mobilisation of arsenic in groundwater used for drinking supplies^25^,^26^. One possible mechanism of DOC release during abstraction is the erosion of biofilms and colloids or sloughing of biofilms from aquifer surfaces due to increased water velocity or shear stress. Research into the effect of water velocity and shear stress on colloid stability and biofilm erosion or sloughing has been limited to drinking water supply pipelines^27^,^28^ and assessment of the effects on porosity and pore scale flow and diffusion caused by biofilm growth and sloughing in sedimentary or unconsolidated groundwater bioremediation systems^12^,^13^,^29^. Knutson, *et al.*^29^ found that shear strength were a significant mechanism controlling biomass development in a porous medium where water was injected for bioremediation purposes.

Shear detachment mechanisms for biofilms resulting from continuous substrate injection associated with bioremediation in porous media has been discussed in detail in MacDonald, *et al.*^17^. Detachment due to shear stress was found to reduce biomass by an order of magnitude and it was concluded that shear detachment played an important role in redistributing biomass within the aquifer^17^. Rittmann^30^ and Rittmann^31^ discuss the effects of shear stress on biofilm detachment within bioreactors using different reactor mediums such as fluidised sand and gravel beds. It was noted that biofilm loss rate due to shear stress was influenced by the composition of the porous medium with losses being higher on smooth surface sands relative to activated carbon. In this instance we are testing the effects of abstraction only within a fractured rock aquifer with no water or solute injection. We expect that shearing of biofilms would in this scenario play the major role in DOM mobilisation.

In this study we aim to identify changes in DOC characteristics and concentrations within a highly stressed aquifer. Groundwater levels and velocities were fluctuated by groundwater abstraction experiments at rates above the viable pumping rate. Through a combination of direct measurements of water, aquifer matrix and overburden OC we aim to determine whether bound organic carbon (OC) is released as a result of the change in aquifer conditions. Quantification of DOC is undertaken by conventional laboratory methods^32^,^33^ and characterisation of DOC is undertaken through measurement of optical fluorescence and absorbance^1^,^34^,^35^. The experiments provide insights into changes in the concentration and character of groundwater DOC as a result of perturbations caused by groundwater pumping. The analysis of the variations in DOC within an aquifer during an abstraction experiment has not been undertaken before.

The experiment was undertaken at the UNSW Wellington Research Station in the central west of NSW, Australia (Fig. 1). See Graham, *et al.*^36^ for further information on the site. The research site is located on a gentle slope of less than five degrees with a Devonian fractured basalt/metasediment rock aquifer overlain by residual soils (between 0.5 and 9 metres thick) and flanked by a deep (up to 20 metres below ground level (mbgl)) Quaternary alluvial channel approximately 50 metres downgradient from the groundwater abstraction location. The fractured rock system outcrops in the upper reaches of the slope (approximately 100 metres south of the bore field) and becomes deeper down the slope to approximately 7 mbgl at the abstraction location.

Based on observations made during drilling (December 2011 to February 2012) and head levels before and during abstraction the groundwater system in the vicinity of the bores is believed to consist of two fracture zones: a lower and upper fracture zone. Connectivity between these systems is moderate to low with both aquifers having similar piezometric heads under equilibrium conditions, however the upper system demonstrated a lagged response (e.g. drawdown) to abstraction from the lower system. The upper fracture system was overlain by weathered bedrock and saprolite. The upper system is therefore considered to be directly recharged by infiltrating rain water (Fig. 2).

## Results

Figure 3 presents a summary of the experimental results including pumping rates, measured groundwater level and DOC concentration response for boreholes BH27s, BH27d and BH45. Pumping rates varied between 12 and 30 m^3^/hr (Table 1). Approximately 410 m^3^ of water was abstracted over the five day experiment. The abstraction was sufficient to pump the aquifer in the immediate vicinity of the abstraction well dry once or twice a day. The abstraction was also observed to reduce groundwater levels 5 m distant from the abstraction bore by up to 7.5 m elevation.

Prior to, during, and following the experiment, chemical characteristics (temp, pH, EC and DO) of the water were measured in the field. These have been summarised in Table 2.

### Sedimentary Organic Carbon

**Figure 2:**
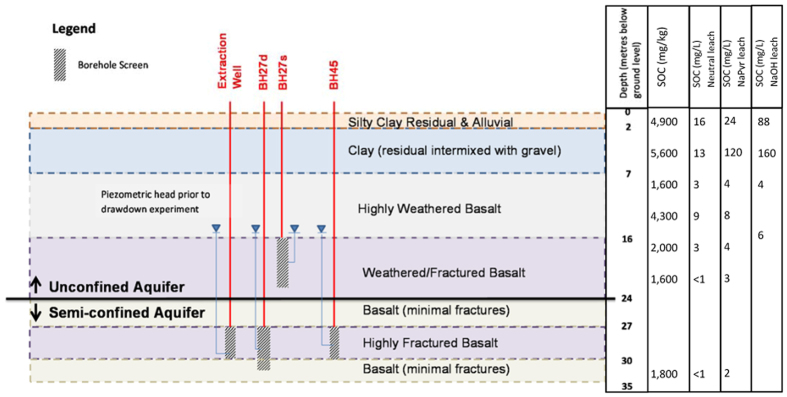
Schematic cross section of the investigation area.

The SOC content within the stratigraphic profile as measured from drilling samples is shown on Fig. 2 and Table 3. The results show that the amount of SOC leached into distilled water is generally low (~2 mg/L) within the deeper (>7 mbgl) sediments, however elevated concentrations (up to 160 mg/L) were present within the soil profile, suggesting a potential DOC source which could be mobilised by infiltrating water. The location of this elevated SOC within the stratigraphic profile (4-5 mbgl) is vertically removed from the saturated zone of the groundwater system (approximately 16 mbgl) and apart from slow long term leaching with infiltration events this carbon is unlikely to be mobilised in the short term by the groundwater abstraction experiment (note there were no rainfall events during the abstraction experiment). In general the leachable fraction by any of the leaching methods is only a tiny percentage (2.8%) of the total SOC in the profile.

### Variation in Dissolved Organic Carbon due to Abstraction

An increase in DOC concentration was identified as a result of the high intensity pumping (i.e. abstraction rates which exceeded the aquifers yield). The highest DOC concentration the upper fracture zone (72.75 mg/L) was detected between one and seventeen hours after the abstraction well had been turned off and the aquifer left to recover. The highest DOC concentration in the lower fracture zone (14.32 mg/L) occurred between one and six hours after the initial abstraction commenced (Fig. 4).

**Figure 3:**
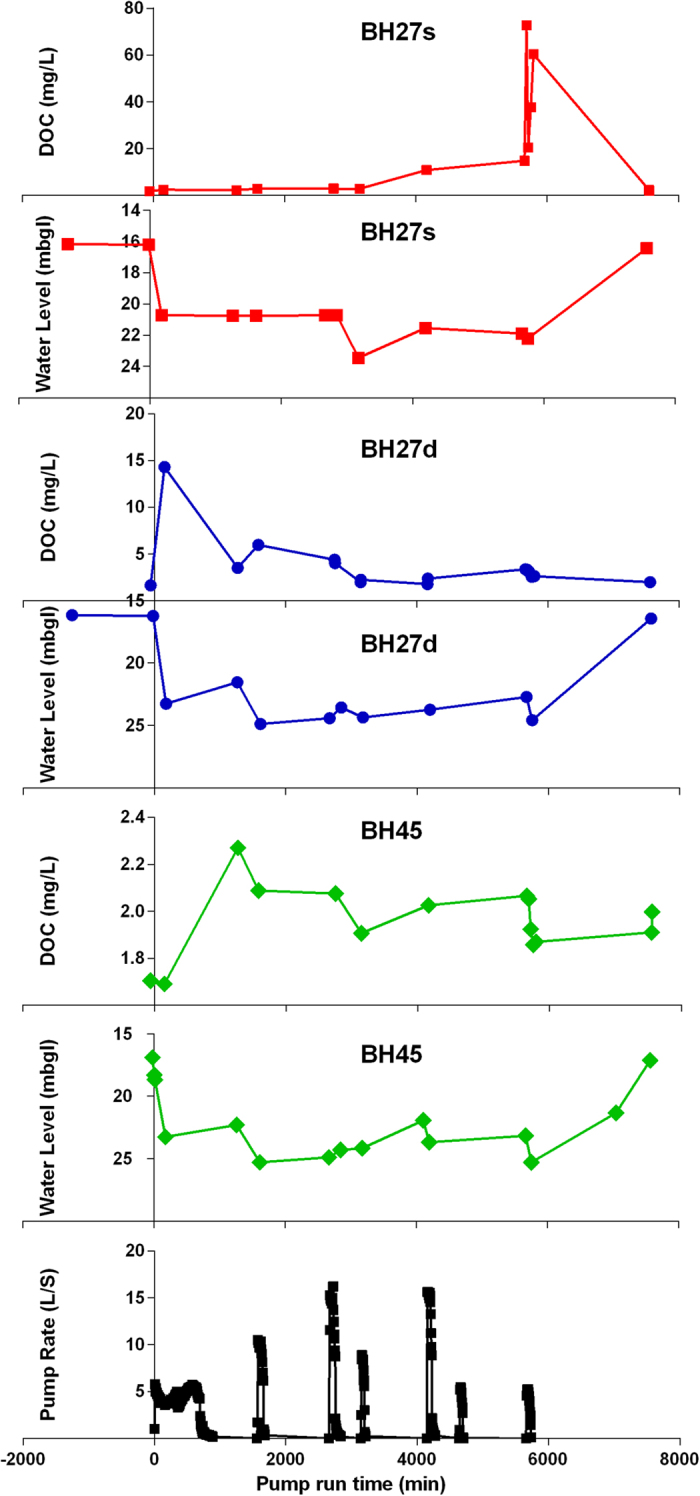
Pump rate and time series of groundwater levels and DOC.

The measured initial DOC concentrations in the upper aquifer (Borehole BH27s – Fig. 3) were approximately 2 mg/L. Following abstraction these concentrations increased to an average (during the 5 day experiment) of 36 mg/L with a peak of 72.75 mg/L. In the lower fracture zone (Boreholes BH27d and BH45 – Fig. 3) the initial DOC concentrations were approximately 1.6 mg/L, during pumping these concentrations were found to increase to an average concentration (during the 5 day experiment) of 3.98 mg/L with a peak of 14.32 mg/L.

### Fluorescent Dissolved Organic Material

**Figure 4:**
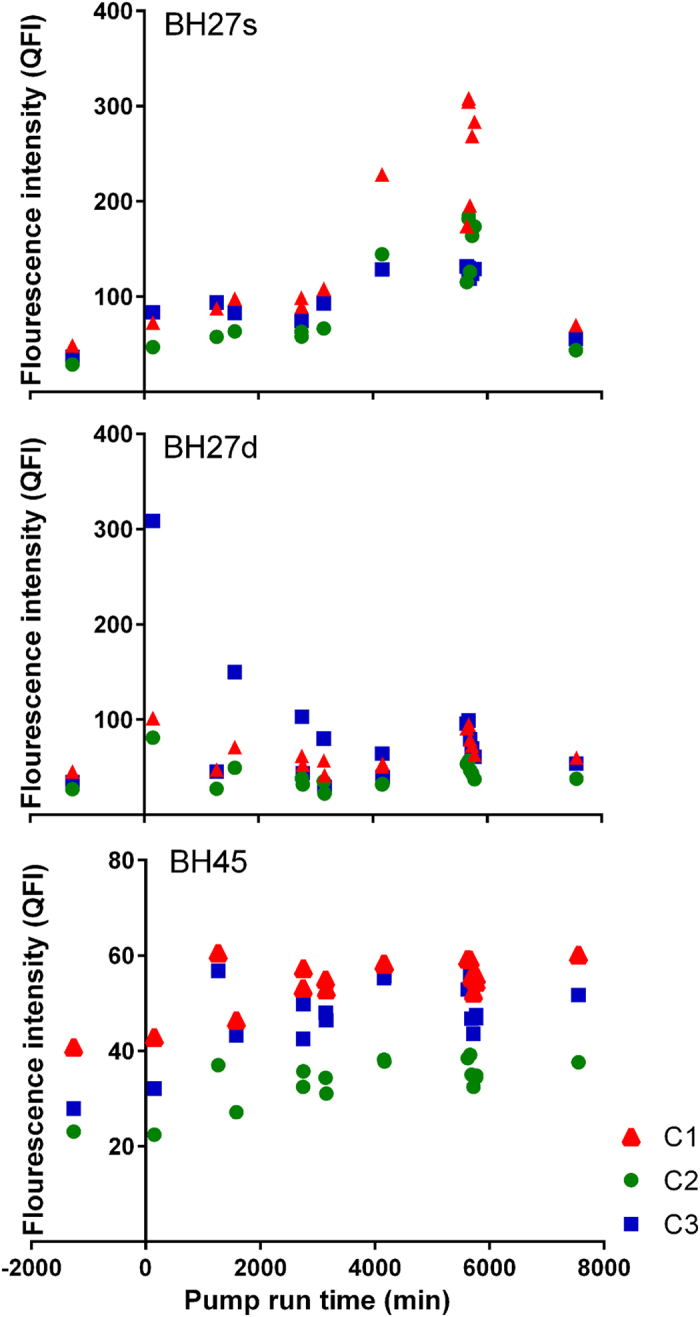
Time series of PARAFAC fluorescence components.

Analysis of the FDOM results using a PARAFAC model identified three consistent components in the data (detailed description of the PARAFAC Model is contained in the methods section of this manuscript). The three components were C1-320/400 Ex/Em (Excitation-Emission wavelengths) (terrestrial derived humic material), C2 – 250/470 Ex/Em (reprocessed, allochthonous humic materials) and C3 – 275/350 Ex/Em (microbial/protein type materials), corresponding to peaks M/C, C^+^ and T respectively, as described by Coble^34^ and Ishii and Boyer^37^. Figure 4 shows the quantitative fluorescence intensity of each component in each borehole during the pumping experiment, Table 4 shows the fluorescence intensity of each component, absorbance (at 340 nm) and DOC measured throughout the experiment.

The PARAFAC model results identified increases of up to 594, 624 and 361 percent of components C1, C2 and C3, during the pumping period in the upper aquifer. In the lower fracture zone increases of up to 232, 313 and 851 percent of components C1, C2 and C3 were identified. In this lower zone increases were generally observed immediately following a pumping event and generally returning to initial concentrations between one and sixteen hours after pumping was stopped (Fig. 4). In the lower fracture zone the presence of the C3 component was increased (average increase of 180 percent in borehole BH27d and BH45) with C3 being the dominant component in borehole BH27d (66, 42 and 87 average Quantitative Fluorescence Intensity (QFI) for components C1, C2 and C3 respectively) and being the secondary component (to C1) in BH45 (54, 33 and 46 average QFI for components C1, C2 and C3 respectively).

The correlation coefficients for Components C1, C2 and C3 to DOC in BH27s (Table 5) show a significant correlation between the C1 and C2 components and there is not a significant correlation to the C3 component, however in BH27d there is a significant correlation between C2 and C3. This suggests the FDOM in BH27s is more typical of primary, soil-derived humic material, while the FDOM in BH27d is more typical of microbial or bacterial derived material. The correlation coefficients for BH45 are not significant due to the low variability in DOC at that location.

### DOM Absorbance and the Absorbance/Fluorescence Ratio

Stewart and Wetzel^38^ identified a relationship between the solubility of OM and the ratio of fluorescence intensity to absorbance, with a higher ratio indicating increased hydrophilicity (increased solubility). This relationship was further defined by Belzile and Guo^39^ through additional application of the method to OC adsorbed to colloidal matter. Organic material with higher molecular weight was shown to absorb light strongly at 250 nm, but only fluoresced weakly, while OC of lower molecular weight fluoresced more intensely per unit absorbance. Baker, *et al.*^40^ demonstrated that peak C (excitation 300–350 nm): absorbance (340 nm) ratio had a high correlation (0.86) to hydrophilicity of DOC.

**Figure 5:**
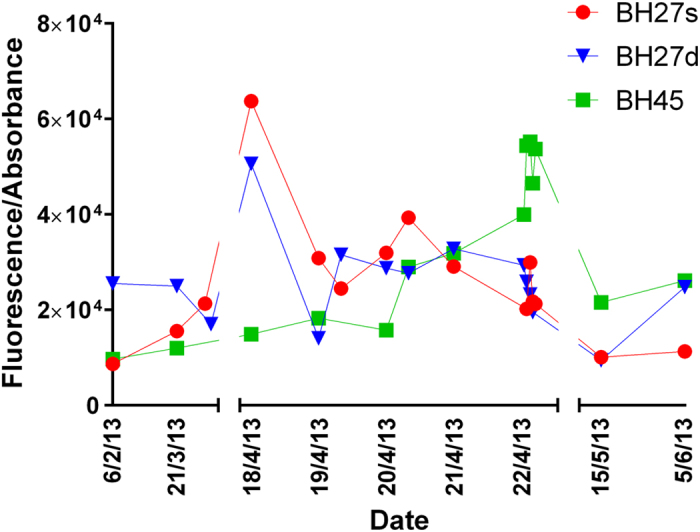
Ratio of total fluorescence to absorbance (340 nm) in boreholes prior to, during and after pumping.

Figure 5 shows the ratio of fluorescence intensity to absorbance for each of the borehole samples one month before, during, and one month after the experiment. The ratios before and after are similar in boreholes BH27d (pre abstraction- 25,514 to post abstraction 26,126) and BH27s (pre-abstraction 9,700 to post abstraction 11,285). An increase was noted in borehole BH45 (pre-abstraction 9,700 post abstraction 26,125). An increase (peak percentage increase of 657, 198 and 561 for Boreholes BH27s, BH27d and BH45 respectively), but with significant variability, is observed during the abstraction events. The ratio measured in all boreholes increased during the experiment (maximum ratios of 63,750, 50,611 and 54,393 for Boreholes BH27s, BH27d and BH45 respectively). The increased FDOM and chromophoric DOM (CDOM) being measured is more hydrophilic or has a lower molecular weight^38^. Reviewing the longer term effects of the experiment shows that the ratio at borehole BH45 increases throughout and following the experiment, suggesting that the character of FDOM/CDOM present in this location consistently changes to a more hydrophilic or lower molecular weight FDOM/CDOM type. The ratio in boreholes BH27s and BH27d are more stable. Borehole BH27s shows a general decreasing trend following the experiment, while borehole BH27d initially decreases however, it recovers to pre-experiment values within two months of the experiment. The decreasing ratios suggest increased hydrophobicity/higher molecular weight FDOM/CDOM character is present in these locations during that time.

## Discussion

Here we have undertaken a study of the effects of high intensity abstraction of groundwater on OM mobilisation within a fractured rock aquifer. Previous studies in this research area have focussed on injection of water into aquifers and its effects on biofilm growth and the resultant variations in aquifer conductivity. We consider it likely that during periods of hydrological equilibrium the majority of organic carbon within the aquifer is adsorbed to solid surfaces as sedimentary organic carbon. During high intensity abstraction, high velocity groundwater flow sloughs and erodes biofilm from aquifer surfaces and increases the DOC concentration in the immediate vicinity (within 5 metres in this experiment) of the abstraction well.

Measurement of leachable SOC with de-ionised water, sodium hydroxide (NaOH) and sodium pyrophosphate (NaPyr) leaching mediums demonstrated that significant amounts of leachable OC were not present in the overlying sediments and the largest component of SOC present was in a stable form. Based on the generally oxidised state of the groundwater at the site (Table 2) it is considered unlikely that lowering the groundwater level has resulted in significant oxidation and aerobic degradation of SOC causing increased leaching of DOC as previously observed by Moore and Dalva^41^. Therefore, although small amounts of leachable DOC may migrate through the soil profile with infiltrating rain water, this component seems relatively minor, which is supported by the study of Borisover, *et al.*^42^.

The physical effect of water velocity on biofilm detachment is broadly categorized into two processes: erosion of small particles from the surface of the biofilm and sloughing of large pieces (detachment from the inner area or base of the biofilm). The effects of variations in shear stress on biofilm detachment within drinking water pipelines and porous media has previously been studied^11^,^17^,^27^,^28^,^29^,^43^,^44^,^45^,^46^. However, to our knowledge it has not yet been studied in fractured rock aquifers. Hence, to gain insights here, we reference the findings of shear stress promoted biofilm detachment in pipe systems and unconsolidated porous sediments. We consider pipe systems more closely replicate likely flow patterns in a fractured rock aquifer than studies in unconsolidated porous sediments. Choi and Morgenroth^28^ found that constant shear stress resulted in erosion type detachment, while a sudden increase in shear stress caused significant increases in both the concentration and particle size of the detached biofilm. Cloete, *et al.*^27^ analysed the response of biofilm detachment to fluid velocity variations within pipe systems. Their study identified biofilm detaching at velocities of above 3 to 4 metres per second. These detaching velocities were sufficient to result in ongoing erosion of the biofilm preventing further build-up of biofilm layers. Lower velocities were found to be capable of detaching biofilms, but were not sufficient to prevent ongoing build up. Wang, *et al.*^43^ noted that biofilms grown in low velocity regimes were more likely to detach and measured detachment rates of between 40 and 60 percent during flows ranging between 0.04 and 0.28 metres per second. A value of 0.005N/m^2^ was suggested to best represent the critical shear stress, at which biofilm is detached in an average porous medium for various microbial species^12^,^29^.

The velocity of water flowing through a fractured rock aquifer is potentially highly variable and dependent on flow events (i.e. abstraction) and on the connectivity of the fracture system. It is a common assumption when assessing fractured rock aquifers to ignore the matrix flow and focus only on flow within the fractures^47^. Approximate calculations of possible flow velocities within fractures in the immediate vicinity of the abstraction bore have been made based on the peak abstraction rate of 30.81 m^3^/hr. The surface area of flow at the abstraction well aquifer boundary would be 2.2 m^2^ (based on a screen radius of 0.12 m and screen length of 3 m). Flow across this surface area would be delivered by the permeable fractures which based on field observations (made during installation of the abstraction bore) and review of core material collected from an adjacent borehole (see supplementary material) make up between 0.1 and 5 percent of the aquifer matrix, the actual surface area of flow would therefore then be reduced to between 0.0022 and 0.11 m^2^. Using the principle of continuity of flow^48^:


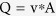


(where Q = flow in m^3^/s, v = velocity in m/s and A = area in m^2^), the velocity expected within the fractures would be between 0.38 and 0.08 m/s. However, it is reasonable to assume that at times during the peak abstraction rates flow would be limited to only a few fractures representing less than one percent of the total rock mass. In this instance the flow velocity in these fractures are likely to exceed 0.4 m/s. This flow rate could be even higher during short pulses due to non-linear responses within the fracture matrix as the pump is switched on or off.

Novakowski^49^ suggested that in most natural systems a few fractures deliver a majority of the flow resulting in very high velocities within these fractures. This results in a zone of non-Darcian flow in the aquifer near the well and non-linear responses to pumping which are considered to be a result of a large transition zone between linear and turbulent flow. This transition zone is caused by fracture geometry characteristics including fracture roughness, dead end voids, aperture variations and contact area^49^. The water velocity is expected to decrease with distance from the extraction bore due to a radial increase in fracture density. This increase is dependent on fracture connectivity and other factors as described by Novakowski^49^. At a distance of four metres from the extraction bore (borehole BH27d) the fracture flow area is expected to increase from 0.075 m^2^ to 0.38 m^2^, resulting in flows of between 0.11 m/s to 0.02 m/s. These flow rates are considered sufficient to result in the observed biofilm detachment at borehole BH27d. Inspection of the core materials from the fractured rock aquifer suggest that fracture surfaces at the subject site were smooth and would therefore be expected to facilitate significant biofilm loss due to shear stress.

The estimated shear velocity at the subject site was considered during abstraction to be within the range of 0.40 to 0.08 m/s, if the water density is assumed to be 1 g/cm^3^ then the shear stress can be expressed as 0.4 to 0.08 N/m^2^. This is well within the range of 0.005 N/m^2^ suggested by Tang, *et al.*^12^ and Knutson, *et al.*^29^. As outlined above, the shear stress required to detach biofilm within water pipes, are generally a magnitude higher than those required for detachment in porous mediums and would be considered more relevant to what may be expected in a fractured rock scenario. In either case the study has shown that shear velocity resulting from the high intensity abstraction could be expected to exceed the required biofilm detachment shear velocity.

Further complicating the potential for SOC to mobilise during abstraction is the concept of fracture skins, described as a thin coating on the rock matrix with different sorption and diffusion properties to that of the undisturbed rock matrix. The skin includes zones of altered rock and coatings of the rock surface by organic matter, precipitated minerals and infiltrated debris^50^ and their influence as a potential source of DOC^51^.

van Beek, *et al.*^52^ compared variations in sediment particle volume and size distribution in groundwater abstracted at varying rates. Their experiment found that higher discharge rates corresponded to increased particle volume and particle size (from 2 to 10 μm). It was also noted that the concentration of particles was higher in younger wells suggesting that ongoing abstraction will eventually exhaust the accessible particle supply around the abstraction well. The van Beek *et al.*^31^ experiment was undertaken within an alluvial aquifer and therefore differs in geological setting (and hence flow patterns) from our experiment, however a similar phenomenon is expected to occur within a fractured system.

Considering the rate of abstraction achieved during the experiment (up to 0.28 metres per second), the resulting shear stress and water velocity would be sufficient to result in sloughing of biofilms with potential to include both particulate SOC and DOC^43^ from the fracture surfaces. This is demonstrated by elevated concentrations of DOC in borehole BH27d at the start of the abstraction, which were identified as FDOM with an elevated ratio of PARAFAC model Component 3 to Components 1 and 2. Component 3 is representative of the microbial/protein type DOM which are expected to be the primary type of DOC released from biofilms^43^. During the experiment it was noted that the DOC increase in BH27d was higher than that measured in BH45 and that the DOC identified in BH45 was typically of lower molecular weight. This is expected to be due to the proximity of piezometer BH27d to the abstraction point and exposure of fractures close to the extraction well to higher water velocities and thus higher shear stresses.

Stewart and Wetzel^38^ and Belzile and Guo^39^ identified a relationship between the molecular weight of dissolved humic material and the fluorescence to absorbance ratio. These correlations were utilised to establish any variations in the relative molecular weight of the DOC identified (Table 4, Fig. 5). The DOC observed in BH45 was noted to have a relatively low molecular weight and is likely to be composed of relatively hydrophilic OC. We hypothesise that this is more mobile and is carried by groundwater being drawn in from other areas of the aquifer. The measured long term trend suggested that the relative molecular weight of DOC within borehole BH45 continued to decrease after the abstraction experiment ended. This suggests that the experiment had acted to mobilise relatively higher molecular weight OC in the immediate vicinity of the abstraction well (borehole BH27d) by the velocity shearing action. As for the increase in the relatively lower molecular weight fraction it is possible that by lowering the water table we are sampling more recently recharged waters from across the shallower part of the general aquifer.

The elevated concentrations of DOC identified during the abstraction experiment in borehole BH27s was mainly due to increase in the C1 to C2 components, which are indicative of locally derived humic and reprocessed humic materials. Borehole BH27s is screened across the upper fracture zone which is likely to receive direct recharging waters which have percolated through the overlying soil/saprolite profile.

This experiment has found that available DOC in a groundwater system in a normal state (not exposed to anthropogenic abstraction) would generally be within the range of 1–2 mg/L. We assume this is due to OC entering the system being adsorbed, taken up by microbes or microbially processed (mineralised). However, increased groundwater velocities in fractures during abstraction resulted in larger shear stresses within the aquifer. These two factors are considered to have contributed to increased DOC mobilisation of up to 72 mg/L within the aquifer.

Assessment of SOC within the geological profile has not identified a significant source of OC within the vadose or unsaturated zones as the SOC in these locations is in a stable (non-leachable) state. We consider it likely that, greatly increasing the water velocity and hence the shear stress in the immediate vicinity of the abstraction bore would result in the shearing of biofilms from aquifer surfaces or fracture skins^27^,^28^. This plausible explanation is supported by the observed increase in relatively high molecular weight OC due to the pumping, which in the absence of leachable OC higher in the profile must result from biofilm detachment.

In our study the effect was observed within bores four metres from the extraction well but not at 10 metres, however more generally this distance would in other settings be dependent on the fracture density and abstraction rates. While van Beek, *et al.*^52^ have demonstrated that the colloid supply may be exhausted over time, there would be ongoing accumulation of biofilms on surfaces within the aquifer as observed in water supply pipes in Cloete, *et al.*^27^. This process would continue to concentrate OC from the groundwater in a form that can be reintroduced into the terrestrial cycle through groundwater abstraction.

Future research could assess whether the release of DOC during abstraction is limited to the areas immediately surrounding the abstraction well or if DOC further from the abstraction well would also be released as the abstraction continues as this would determine the potential quantity of carbon released during abstraction. We have also suggested that one of the main sources of OC (biofilms) could replenish and therefore could act as an ongoing source of DOC within the groundwater. Assessment of the potential for the biofilms to replenish requires confirmation. A long term study of the steady state OC flux during abstraction would therefore be a useful study for comparison. Finally, the sloughing of biofilms was identified as a likely source of DOC. Of interest would be future studies incorporating both particulate and dissolved organic carbon analysis from samples of groundwater collected during varying stages of abstraction.

## Methods

Abstraction was undertaken from an abstraction well (EW02) cased in 240 mm polyvinyl chloride (PVC) and a four metre screen was placed across a two to three metre fracture zone located at a depth of approximately 28 to 30 mbgl. Monitoring was undertaken in borehole BH27, a nested well located approximately four metres to the south of the abstraction well and BH45 located approximately 10 metres south west of the abstraction well. The shallow piezometer in BH27 (BH27s) was constructed of 50 mm PVC with a machine slotted screen between 20 and 23.5 mbgl, intercepting a minor fracture zone within weathered bedrock. The deep piezometer in BH27 (BH27d) was also constructed of 50 mm PVC with a machine slotted screen between 27.5 and 33 mbgl, intercepting the same deeper fracture zone as EW02. A one metre thick bentonite plug was placed in the BH27 borehole between 25 and 26 mbgl to isolate the two piezometers. Borehole BH45 was cased with steel to 3.5 mbgl but uncased to a depth of 30 mbgl. During drilling of BH45 the upper fracture zone identified in BH27 was not encountered, however a deeper fracture zone was encountered at a depth of approximately 26 to 27 mbgl.

As part of a separate project a borehole (borehole BH25) was drilled approximately 50 metres to the east of borehole BH27. Borehole BH25 was diamond cored through the same geological materials as boreholes BH27 and BH45. The core samples were inspected to determine the representative fracture distribution and size. Photos of this core material have been included in the supplementary material.

During drilling of boreholes BH27, BH45 and extraction well EW02 it was noted that the bedrock between 23.5 mbgl and 27 mbgl increased in competency and was dry, suggesting connectivity between the two fracture zones would be limited to fracture flow. Prior to abstraction groundwater levels in in all boreholes were generally 16.10 to 16.20 mbgl. During abstraction an immediate drawdown response was noted in boreholes BH27d and BH45, however the response in BH27s lagged by approximately an hour suggesting that the upper and lower fracture systems are connected, but with limited connectivity.

Throughout the experiment the groundwater abstraction rates were monitored with a V-notch weir and groundwater levels were recorded every 10 min using submersible pressure transducers. Pumping rates and sampling times are shown in Table 1. Abstraction was undertaken at a rate which exceeded the yield of the well and EW02 was pumped dry one to two times a day for five days (18 April to 22 April 2013 – Table 1). This cyclic operation of the pump allowed for an aggressive abstraction schedule that promoted variations in water velocity and shear stress in the aquifer to allow potential biofilm detachment to be incorporated into the measured DOC mass.

Groundwater samples were collected monitoring wells within the lower fracture system (Boreholes BH27d and BH45) and upper fracture system (BH27d) using a centrifugal pump one month, a week and a day prior to the experiment and then during the experiment approximately one hour after abstraction had commenced (Table 1 details the sampling times). The sampling pump intake point was positioned at the approximate depth of the main fracture zones as identified during piezometer installations, these were 29 mbgl for BH27d, 20 mbgl for BH27s and 28.5 mbgl for BH45.

Samples were then collected one day, one week and one month following the experiment. Prior to sampling all locations were dipped to measure the standing water level. Groundwater was pumped through a flow cell measuring pH, EC, DO and temperature. Measurements were made using an Orion Star A329 multi parameter water quality meter which was calibrated on a daily basis. Prior to each sample three well volumes were purged from the monitoring wells. During the experiment the wells were again purged and samples were collected following stabilisation of the field chemical characteristics. During the abstraction event on 22 April samples were collected every half hour over a ninety minute period.

Throughout this study we have used different terms for different types or states of organic carbon for clarity our interpretation of the terms is defined here; SOC – sedimentary or total amount of organic carbon in soil or sediment as measured by standard laboratory methods), DOC (portion of organic carbon [including chromophoric and fluorescent organic matter measured as FDOM] within water which passes through a 0.45 μm filter.

Prior to its first use, a sample of the groundwater pump tubing was soaked in distilled water and a sample of the soak water was subjected to all analysis methods to ensure it would not be a source of DOC or fluorescent material. Water samples were field filtered using 0.45 μm filters, collected in acid washed plastic containers and stored in an ice chest until analysis. Analysis for DOC was undertaken within five days of sampling by the UNSW Analytical Centre. The samples were acidified offline with 0.1 ml of H_3_PO_4_ per 9.9 mL of sample and purged (using nitrogen) to eliminate the inorganic carbon interference. The treated samples were then measured for DOC by a TOC Analyser (Aurora 1030 wet oxidation TOC analyser, OI Analytical, College Station, TX, USA) as per method 5310D^32^.

Subsamples for fluorescence excitation/emission matrix (EEM) spectral analysis were also measured on the field filtered samples using an Horiba Aqualog Spectrofluorometer (ASF). Scans were conducted using excitation wavelengths from 240 to 400 nm at 10 nm steps. Emission wavelengths were collected from 210 to 600 nm at 2 nm bandwidth and 1s integration time. The intensity of all EEM spectra was normalised by dividing the integrated intensity area of the Raman water curve at 350 nm wavelength excitation from the 370 to 450 nm emission wavelength range to give a quantitative fluorescence intensity (QFI)^53^. Inner filter correction was undertaken and scatter lines masked.

The EEM data was then incorporated into an existing PARAFAC model which had been developed utilising 480 EEMs from the wider Wellington Research Station field site (Graham *et al.* 2015^36^) using methodology described by Leurgans and Ross^54^, Bro^55^ Stedmon and Bro^56^ and Chen, *et al.*^57^. A three component model was derived by applying the PARAFAC method to the 480 EEMs. The three component model had a core consistency of 85, a four component model was developed, however core consistency dropped significantly to 59 indicating an over-specified model^58^. The three components were characterised by the following ranges: C1 – 300–350/400–450 nm Ex/Em and <260/400–450 nm Ex/Em, C2 – 240–270/430–500 nm and 360–400/ 430–500 nm Ex/Em and C3 – 270–280/350 nm and <250/350 nm Ex/Em, corresponding to peaks M/C (terrestrial derived humic material), C^+^ (reprocessed, allochthonous humic material) and T (tryptophan – microbial/protein like) respectively as described by Coble^34^ and Ishii and Boyer^37^. Absorbance data was also collected for a wavelength of 340 nm using the ASF.

Selected soil and sediment samples were collected directly from the drill cuttings during installation of the groundwater wells. These samples were placed directly into air tight plastic bags and were stored in a dark container until analysis. Selected samples were analysed by a National Association of Testing Authorities (NATA) accredited laboratory to determine TOC using the Walkley Black method^33^, a trimetric method that measures oxidisable organic content of soils on a one gram soil sample.

To determine the potential leachable quantity of OC within the site soil profile, Australian Standard Leaching Procedures (ASLPs (Australian Standard AS 4439.3 – 1997))^59^ were used at a NATA accredited laboratory. The method was modified through use of three different leaching mediums (de-ionised water, 0.1 molar NaPyr and 0.5 molar NaOH). The method utilised a 300 g mass of soil reduced to pass through a 2.4 mm aperture sieve and then evenly split between the three separate leaching mediums. The concentration of OC in the leachate was then determined using a TOC analyser applying the combustion method 5310B as per Rice, *et al.*^32^.

## Additional Information

**How to cite this article**: Graham, P. W. *et al.* Dissolved Organic Carbon Mobilisation in a Groundwater System Stressed by Pumping. *Sci. Rep.* **5**, 18487; doi: 10.1038/srep18487 (2015).

## Supplementary Material

Supplementary Information

## Figures and Tables

**Figure 1:**
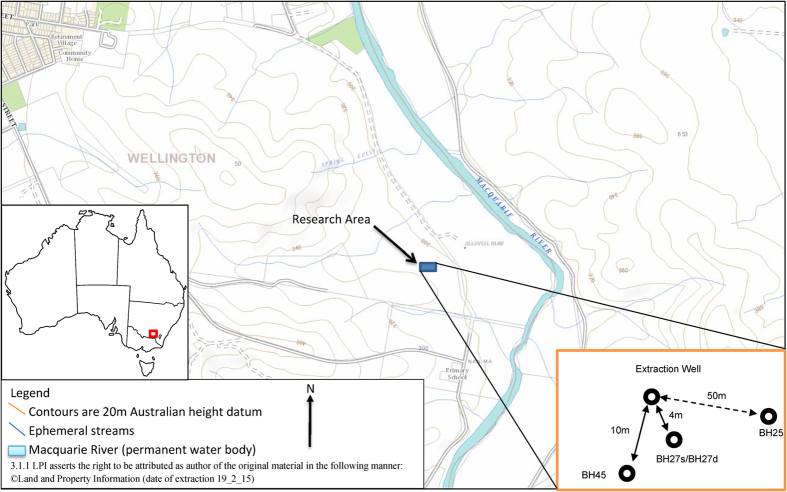
Site location, regional topography and relative bore locations (3.1.1 LPI asserts the right to be attributed as author of the original material in the following manner: ©Land and Property Information (date of extraction 19/02/2015)).

**Table 1:** Pumping Rates and sampling events.

Date	Pump				Sample Time (UTC + 10 hrs)		
	Start time	End time	rate (m^3^/hr)	Total Abstraction (m^3^)	BH27s	BH27d	BH45
21/03/2013	n/a	n/a	n/a	n/a	14:40	15:00	15:30
9/04/2013	n/a	n/a	n/a	n/a	14:22	14:49	15:11
18/04/2013	12:00	03:00*	12.91	193.75	11:20	11:35	11:55
18/04/2013	—	—	—	—	16:00	16:22	16:40
19/04/2013	14:45	16:05	24.45	32.60	09:00	09:25	09:45
19/04/2013	—	—	—	—	15:00	15:25	15:55
20/04/2013	08:30	11:25	25.45	71.31	08:45	09:00	09:18
20/04/2013	16:30	17:30	23.34	23.34	17:05	17:20	17:35
21/04/2013	09:20	11:20	30.81	61.63	08:30	08:45	09:00
21/04/2013	17:30	18:25	12.53	11.48	17:35	17:50	18:12
22/04/2013	10:30	11:43	16.24	19.76	10:00	10:15	10:28
22/04/2013	—	—	—	—	10:45	10:55	11:10
22/04/2013	—	—	—	—	11:20	11:32	11:42
22/04/2013	n/a	n/a	n/a	n/a	11:48	11:55	12:10
22/04/2013	n/a	n/a	n/a	n/a	12:25	12:35	12:48
8/05/2013	n/a	n/a	n/a	n/a	09:03	09:25	09:55
15/05/2013	n/a	n/a	n/a	n/a	14:09	14:28	14:52
5/06/2013	n/a	n/a	n/a	n/a	11:23	11:48	12:06

n/a no pumping.

UTC–Coordinated universal time.

*abstraction ran until 3am on 19/04/2013.

- indicates sampling within the same pumping period, for pump reading see rows above

**Table 2:** Field Chemical Characteristics.

Borehole	Timing	Temp (ºC)	pH	EC (μs/cm)	DO (mg/L)
BH27s	Before	20.5	6.77	719	4.9
	During	19.9	7.21	1,592	n/a
	After	19.6	7.11	859	5.5
BH27d	Before	20.8	6.95	845	7.1
	During	19.3	7.16	925	n/a
	After	19.3	7.01	865	10
BH45	Before	20.8	7.03	777	7.3
	During	20.5	7.12	969	n/a
	After	19.2	7.08	861	9.5

Before = prior to commencement of abstraction (9/04/2013). During = average of measurements taken during abstraction (11 measurements taken between 18/04/2013-22/04/2013). After = measurement taken following abstraction (08/05/2013).

EC – Electrical Conductivity.

DO – Dissolved Oxygen.

n/a – DO probe was faulty for measurements made at these times

**Table 3:** Sedimentary OC analysis results.

	SOC (mg/kg)	Leachable SOC mg/L		
Sample (mbgl)		Distilled Water	Na Pyr	NaOH
BH45 (0.0–1.0)	n/a	16	24	88
BH45 (2.0–3.0)	4,900	<1	11	n/a
BH45 (3.0–4.0)	5,600	7	16	n/a
BH45 (4.0–5.0)	n/a	13	120	160
BH45 (8.0–9.0)	1,600	3	4	n/a
BH45 (9.0–10.0)	n/a	2	4	4
BH45 (12.0–13.0)	4,300	9	8	n/a
BH45 (13.0–14.0)	n/a	5	6	6
BH45 (15.0–16.0)	2,000	3	4	n/a
BH45 (22.0–23.0)	1,600	<1	3	n/a
BH45 35.0–36.0)	1,800	<1	2	n/a
BH27 (0.0–1.0)	n/a	7	36	43
BH27 (1.0–2.0)	2,300	2	10	n/a
BH27 4.0–5.0)	n/a	2	4	4
BH27 (7.0–8.0)	4,300	1	3	3
BH27 (12.0–13.0)	n/a	1	3	2
BH27 (18.0–19.0)	2,200	1	3	n/a
BH27 (21.0–22.0)	1,300	<1	2	n/a
BH27 (22.0–23.0)	1,000	<1	2	n/a

Na Pyr – Sodium pyrophosphate.

NaOH – Sodium Hydroxide.

n/a – no analysis undertaken on this sample

**Table 4:** PARAFAC, absorbance and DOC analysis results.

Date	BH27s					BH27d					BH45				
	C1 (QFI)	C2 (QFI)	C3 (QFI)	α OD (−Log T)	DOC (mg/L)	C1 (QFI)	C2 (QFI)	C3 (QFI)	α OD (−Log T)	DOC (mg/L)	C1 (QFI)	C2 (QFI)	C3 (QFI)	α OD (−Log T)	DOC (mg/L)
21/03/13	54	32	40	0.0087	n/a	44	26	36	n/a	n/a	45	28	38	0.0093	n/a
9/04/13	51	29	36	0.0055	n/a	48	29	45	0.0072	n/a	43	25	30	n/a	n/a
18/04/13 (am)	73	47	84	0.0032	1.90	46	27	34	0.0098	1.67	41	23	28	0.0065	1.70
18/04/13 (pm)	n/a	n/a	n/a	n/a	2.49	102	81	309	0.017	14.32	43	22	32	n/a	1.69
19/04/13 (am)	88	58	94	0.0077	2.29	47	27	46	0.0085	3.53	61	37	57	0.0084	2.27
19/04/13 (pm)	98	64	83	0.0099	2.94	71	49	150	0.0086	5.98	46	27	43	n/a	2.09
20/04/13 (am)	99	63	74	0.0069	3.01	62	38	103	0.0044	4.40	53	32	43	0.0081	2.08
20/04/13 (pm)	108	66	93	0.0068	2.90	57	35	80	0.0034	2.00	53	31	46	0.0045	2.03
21/04/13 (am)	228	145	129	0.0172	10.88	51	32	64	0.0038	1.79	58	38	55	0.0048	n/a
21/04/13 (pm)	n/a	n/a	n/a	n/a	n/a	54	32	40	n/a	2.38	n/a	n/a	n/a	n/a	n/a
22/04/13	174	115	132	n/a	14.91	91	54	96	0.0082	3.37	59	38	53	0.0038	2.07
22/04/13	305	182	128	0.0302	72.75	95	57	99	0.0097	3.32	59	39	56	0.0028	2.05
22/04/13	196	126	119	0.0147	20.50	79	47	79	0.0088	3.11	56	35	47	0.0025	1.92
22/04/13	269	164	124	0.0255	60.63	73	43	69	0.0095	2.63	52	32	44	0.0027	1.87
22/04/13	284	174	129	0.0274	37.64	63	37	61	n/a	2.58	54	34	47	0.0025	1.86
8/05/13	84	50	63	n/a	2.44	64	43	60	n/a	n/a	65	43	59	n/a	1.91
15/05/13	70	44	55	0.0167	2.02	60	38	54	n/a	2.01	60	38	52	0.0069	2.00
5/06/13	n/a	n/a	n/a	0.0237	n/a	n/a	n/a	n/a	0.0066	1.91	n/a	n/a	n/a	0.0065	1.87

n/a – results not available for this sample. QFI – quantitative fluorescence intensity. α OD – UV absorbance at 340 nm

**Table 5:** Correlation Coefficients.

Correlation Coefficient^1^	BH27s	BH27d	BH45	Site Ave^2^
**C1 vs Abs (340 nm)**	**0.82**	0.04	0.07	**0.73**
**C2 vs Abs (340** **nm)**	**0.80**	0.04	0.12	**0.69**
**C3 vs Abs (340** **nm)**	0.47	0.04	0.06	0.37
**C1 vs DOC (mg/L)**	**0.79**	0.38	0.34	**0.86**
**C2 vs DOC (mg/L)**	**0.75**	**0.69**	0.27	**0.84**
**C3 vs DOC (mg/L)**	0.44	**0.94**	0.47	0.28
**Abs (340** **nm) vs DOC (mg/L)**	**0.78**	0.57	0.11	0.56

^1^Correlation coefficient calculated using the Pearson product-moment correlation coefficient method.

^2^Site Ave-average of boreholes BH27s, BH27d and BH45, (n = 12).

^3^bold values are statistically significant.
