# Supplementary material for: Dissolved Organic Carbon Mobilisation in a Groundwater System Stressed by Pumping
Source: Sci Rep. 2015 Dec 22;5:18487. doi: 10.1038/srep18487 (PMC4686979; doi:10.1038/srep18487)
Supplement: Supplementary Information [file srep18487-s1.pdf]

Dissolved Organic Carbon mobilisation in a Groundwater System Stressed by Pumping

P. W. Graham[1], A. Baker, M. S. Andersen

Supplementary Information

Borehole BH25 diamond cores - 6.14 to 31.14 m below ground level (mbgl)

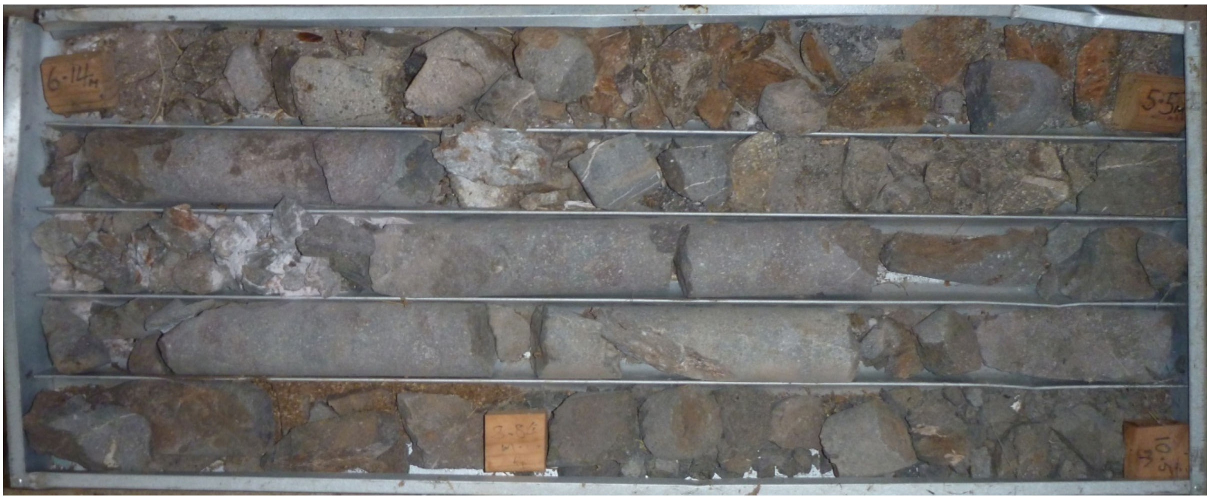

Borehole BH25 diamond cores - 6.14-10.5 mbgl

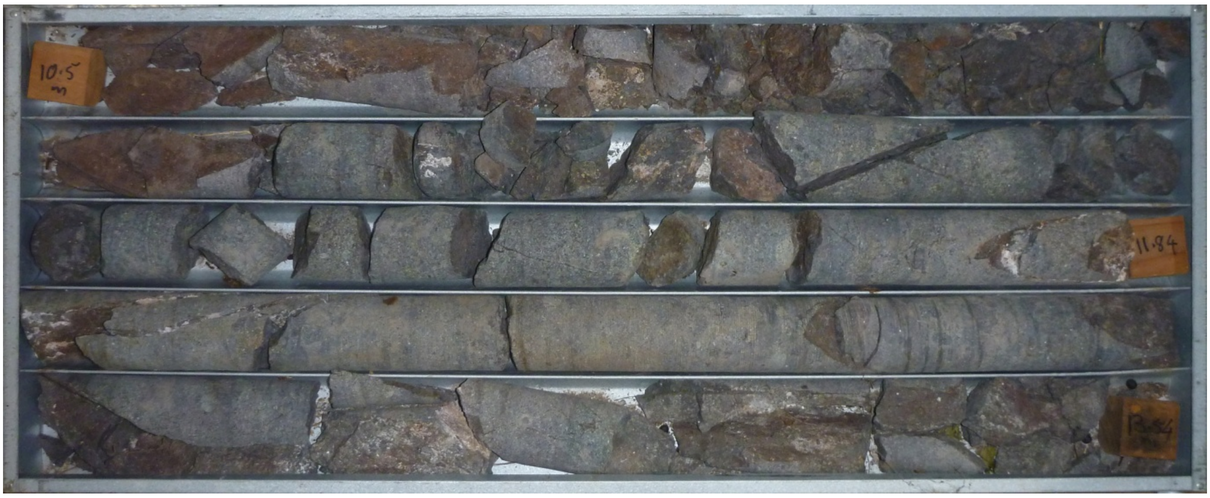

Borehole BH25 diamond cores - 10.5-13.14 mbgl

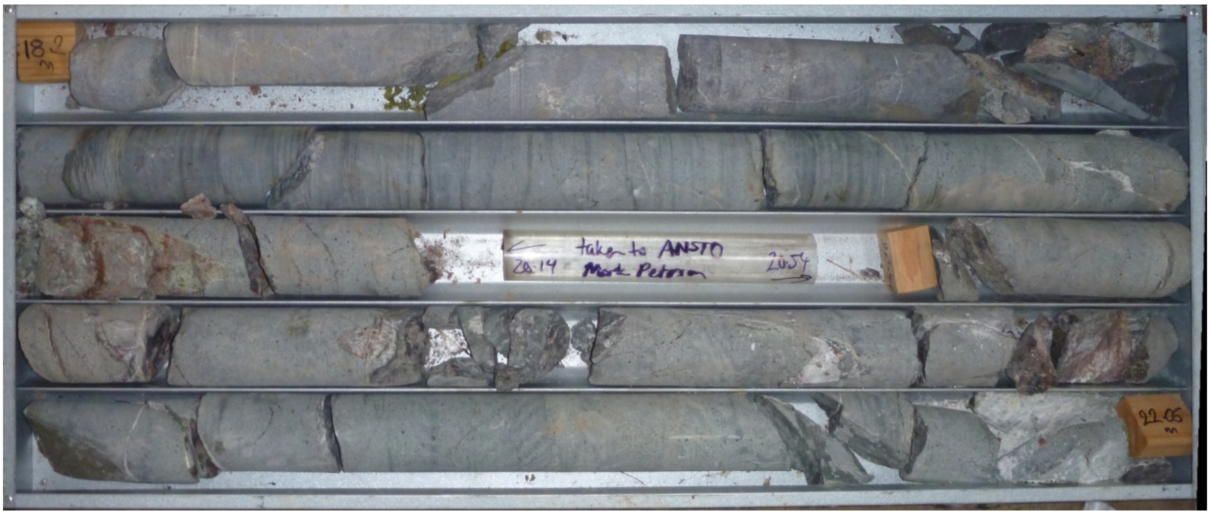

Dissolved Organic Carbon mobilisation in a Groundwater System Stressed by Pumping

P. W. Graham[1], A. Baker, M. S. Andersen

Borehole BH25 diamond cores - 18.2-22.05 mbgl

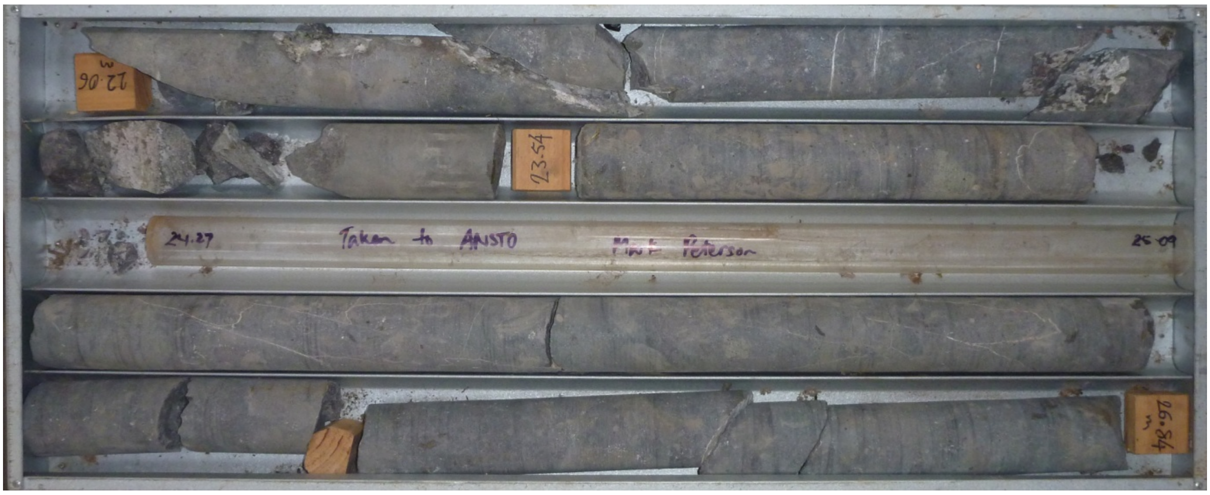

Borehole BH25 diamond cores - 22.05-26.84 mbgl

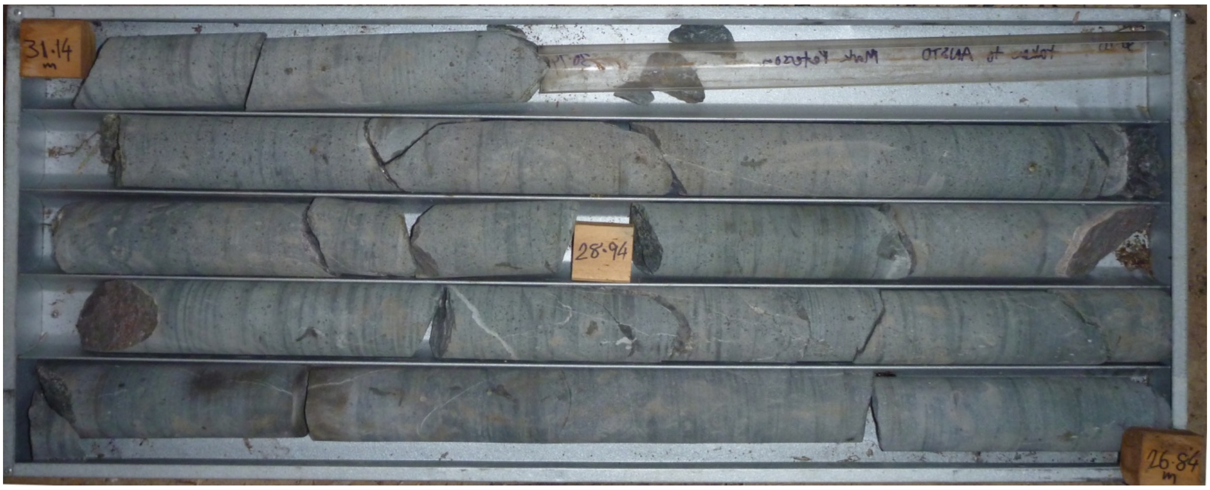

Borehole BH25 diamond cores - 26.84-31.14 mbgl
